# Supplementary material for: Comparison of the Effects of Recombinant and Native Prolactin on the Proliferation and Apoptosis of Goose Granulosa Cells
Source: Int J Mol Sci. 2023 Nov 15;24(22):16376. doi: 10.3390/ijms242216376 (PMC10671185; doi:10.3390/ijms242216376)
Supplement: Supplementary file 1 [file ijms-24-16376-s001.zip › ijms-2683907-supplementary.pdf]

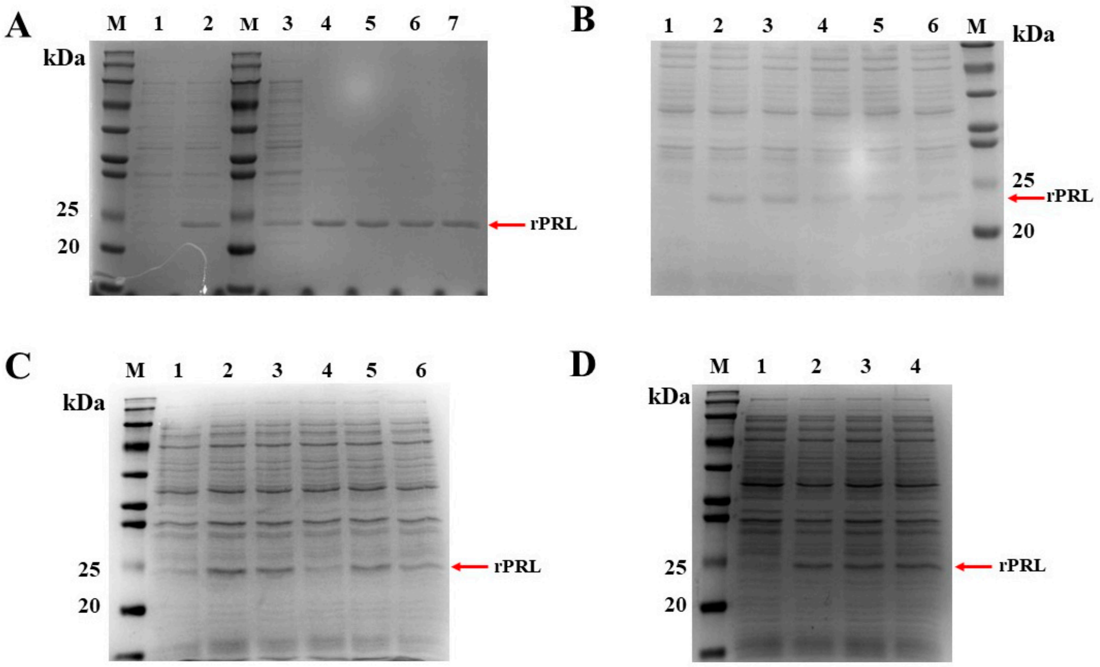

Figure S1. The prokaryotic expression products of pET-28a-*PRL* were obtained from the soluble fraction in E. coli BL21 (DE3) using different optimization conditions. (A) The different concentrations of IPTG are used to induce goose recombinant PRL protein expression. Lane 1, the supernatant after lysis of pET-28a-PRL-BL21 without IPTG; Lanes 2-3, the supernatant after lysis of pET-28a-*PRL*-BL21 with 1mM of IPTG; Lanes 4-7, the supernatant after lysis of pET-28a-*PRL*-BL21 with 0.1,0.25,0.5, or 1mM of IPTG, respectively. (B) The different induction times are used to induce goose recombinant PRL protein expression. Lane 1, the supernatant after lysis of pET-28a-*PRL*-BL21 without IPTG; Lanes 2-6, the supernatant after lysis of pET-28a-*PRL*-BL21 induced with 1mM of IPTG for 4, 6, 8,10, or 12 hours, respectively. (C) The different rotational speeds are used to induce expression of goose recombinant PRL protein. Lane 1, the supernatant after lysis of pET-28a-*PRL*-BL21 without IPTG; Lanes 2-6, the supernatant after lysis of pET-28a-*PRL*-BL21 induced with 1mM of IPTG at the speed of 100, 150, 200, 250, or 300 r/min, respectively. (D) The different temperatures are used to induce the expression of goose recombinant PRL protein. Lane 1, the supernatant after lysis of pET-28a-PRL-BL21 without IPTG; Lanes 2-4, the supernatant after lysis of pET-28a-PRL-BL21 induced with 1mM of IPTG for 15, 25, or 35 ℃, respectively. M, protein marker.
